# Beneficial effects of neuronal ATF6 activation in permanent ischemic stroke

**DOI:** 10.3389/fncel.2022.1016391

**Published:** 2022-10-14

**Authors:** Xuan Li, Ran Li, Liping Lu, Ashis Dhar, Huaxin Sheng, Wei Yang

**Affiliations:** Multidisciplinary Brain Protection Program, Department of Anesthesiology, Duke University Medical Center, Durham, NC, United States

**Keywords:** UPR, MCAO, photothrombotic stroke, long-term, ER stress, proteostasis, transgenic mice

## Abstract

**Objective:** Brain ischemia leads to the accumulation of unfolded/misfolded proteins in the endoplasmic reticulum (ER) lumen and consequently, ER stress. To help cells restore ER function, a series of adaptive stress response pathways, collectively termed the unfolded protein response (UPR), are activated. We have previously demonstrated that the UPR pathway initiated by ATF6 is pro-survival in transient ischemic stroke. However, the effect of ATF6 activation on the outcome after permanent ischemic stroke remains unknown. Here, we addressed this knowledge gap.

**Method:** sATF6-KI mice with functional short-form ATF6 (sATF6) predominantly expressed in forebrain neurons were subjected to two ischemic stroke models: photothrombotic stroke and permanent middle cerebral artery occlusion (pMCAO). Both short-term and long-term functional outcomes were evaluated. Changes in neuroinflammation and cerebrovascular density after pMCAO were also assessed.

**Results:** Compared to littermate controls, sATF6-KI mice performed significantly better in open field, cylinder, and foot fault tests on day 1 or 3 after photothrombotic stroke. However, on days 7 and 14 after stroke, the performance of these functional tests was not significantly different between groups, which is likely related to mild brain damage associated with this stroke model. Thus, to evaluate the long-term effects of ATF6 activation in permanent stroke, we turned to our pMCAO model. We first found that on day 4 after pMCAO, functional outcome was better, and infarct volumes were smaller in sATF6-KI mice vs controls. Next, the 15-day stroke outcome study indicated that compared to control mice, sATF6-KI mice consistently exhibited improved performance in neurologic scoring, tight rope test, and tape removal test, after pMCAO. Moreover, sATF6-KI mice showed higher vascular density and lower activation of both astrocytes and microglia around stroke regions on day 16 after pMCAO.

**Conclusions:** Here, we presented the first evidence that activation of the ATF6 UPR branch is protective in permanent ischemic stroke, which further supports the therapeutic potential of targeting the ATF6 pathway in stroke.

## Introduction

Every year, approximately 800,000 people in the US have a stroke, and about 87% of these strokes are ischemic in nature (Tsao et al., 2022). Treatment for ischemic stroke currently relies primarily on acute thrombolysis and/or thrombectomy to restore blood flow. This reperfusion therapy, however, is accessible to only a fraction of stroke patients. Indeed, for ischemic stroke, the therapeutic window for intravenous thrombolysis is narrow, normally within 4.5 h after the onset of symptoms. While endovascular thrombectomy treatment shows beneficial effects even at 24 h after stroke, only patients with large vessel occlusion and sizeable penumbra are eligible (Saini et al., 2021; Hochrainer and Yang, 2022). Consequently, many stroke patients have permanent vessel occlusion without any effective treatment options. Thus, in the search for cerebroprotective treatments for stroke, both permanent and transient stroke models need to be evaluated.

One promising cerebroprotective strategy for stroke is to leverage cellular self-healing mechanisms that have been refined over time by nature (Yang and Paschen, 2017; Marmolejo-Martinez-Artesero et al., 2021). Among these mechanisms is activation of the unfolded protein response (UPR; Yang and Paschen, 2016; Li and Yang, 2021). The UPR is a key component of the protein quality control network that ensures the integrity and homeostasis of a functional cellular proteome. When misfolded or unfolded proteins accumulate in the endoplasmic reticulum (ER), a major organelle for protein folding/processing of secretory proteins, ER stress ensues, and the UPR is activated. ER stress occurs under pathologic states, such as ischemia, that affect protein homeostasis (proteostasis). Indeed, a large body of evidence has demonstrated that after brain ischemia, drastic changes in the cellular environment (e.g., ATP depletion and Ca^2+^ dysregulation) negatively impact the function and structure of existing proteins, and the synthesis and maturation of new proteins. This leads to the accumulation of unfolded and misfolded proteins in the ER, and activation of the UPR.

The main functions of the UPR are to increase the folding capacity of the ER, degrade potentially toxic unfolded/misfolded proteins, and reduce ER workload by diminishing protein synthesis, concertedly aiming to restore cellular proteostasis and promote cell survival. These functions are fulfilled by three adaptive UPR branches controlled by three ER stress sensors—protein kinase R-like ER kinase (PERK), activating transcription factor-6 (ATF6), and inositol-requiring enzyme-1 (IRE1; Yang and Paschen, 2016; Wang and Yang, 2019). While the PERK branch primarily initiates a translational response to suppress the new synthesis of proteins and thus, reduce the ER workload, the ATF6 and IRE1 branches mainly mediate a transcriptional response by upregulating expression of genes related to protein maturation and degradation, such as ER chaperones and ER-associated degradation (ERAD) components. As the ATF6 pathway has been increasingly considered a promising therapeutic target for ischemia-related diseases (Glembotski et al., 2019), we focused the current study on the ATF6 UPR branch in ischemic stroke.

ATF6 is a 90-kDa transmembrane protein with the C-terminal region present within the ER lumen as an ER stress sensor, and the N-terminal region located in the cytoplasm. Upon ER stress, ATF6 traffics to the Golgi where it is cleaved by S1P and then S2P endopeptidases to generate the N-terminal 50-kDa cytosolic portion of ATF6, also called short-form ATF6 (sATF6). sATF6 has a nuclear localization sequence that guides the translocation of sATF6 into the nucleus where sATF6 acts as a potent transcription factor to regulate gene expression. Both *in vitro* and *in vivo* studies have demonstrated that sATF6-regulated genes are mostly related to adaptive and pro-survival functions in cells (Glembotski et al., 2019). These include ER resident chaperones (e.g., *Grp78*), folding catalysts [e.g., protein disulfide isomerase (*PdI*)], and ERAD components (e.g., *Hrd1*). These genes are also upregulated in neurons after activation of ATF6 signaling (Shen et al., 2021). As expected, the majority of published studies have indicated that the ATF6 pathway is pro-survival (Glembotski et al., 2019). For example, cardiac-specific activation of the ATF6 pathway in a transgenic mouse line exerts remarkable protection against heart ischemia/reperfusion injury (Blackwood et al., 2019).

To examine the role of ATF6 signaling in the brain, we previously developed a conditional and tamoxifen-inducible sATF6 knock-in mouse line, sATF6-MER (Yu et al., 2017). Based on this mouse line, we then generated sATF6-MER; Emx1-Cre (sATF6-KI) mice to restrict sATF6 expression to neurons. Using sATF6-KI mice, we have demonstrated that activation of the ATF6 pathway in neurons protects the brain after cardiac arrest or transient ischemic stroke (Yu et al., 2017; Shen et al., 2021). However, the effect of ATF6 activation on outcome after permanent ischemic stroke remains unknown. This is a critical question, as stroke with reperfusion and stroke without reperfusion have different pathophysiologies (Ma et al., 2020). Indeed, many novel therapies have shown protective effects in a transient stroke model but failed in a permanent stroke model (Shimazu et al., 2005; Berger et al., 2008; Kim et al., 2015). Thus, given the large number of stroke patients with permanent vessel occlusion as well as the encouraging data for ATF6 activation in transient brain ischemia models, this study was designed specifically to examine the effects of ATF6 activation in permanent stroke. We subjected sATF6-KI mice to two permanent stroke models: photothrombotic (PT) stroke and permanent middle cerebral artery occlusion (pMCAO). In both models, sATF6-KI mice showed improved stroke outcome compared to controls.

## Materials and Methods

The protocols for animal experiments were approved by the Duke University Animal Care and Use Committee. All studies were conducted in accordance with the United States Public Health Service’s Policy on Humane Care and Use of Laboratory Animals, and ARRIVE guidelines. All animals had free access to food and water in a room maintained on a 14/10-h light/dark cycle. Animals were randomized into groups using an online tool, and all evaluations were performed by experimenters who were blind to genotypes.

### Animals

C57Bl/6 mice were purchased from The Jackson Laboratory (Bar Harbor, ME). The conditional and inducible sATF6 knock-in mouse line, sATF6-MER, was maintained in the lab (Yu et al., 2017). Similar to our previous study (Yu et al., 2017), we crossed this line with Emx1^Cre/Cre^ mice (JAX stock #005628; C57Bl/6 background) to generate sATF6-MER; Emx1-Cre mice (sATF6-KI). In sATF6-KI mice, sATF6 is FLAG-tagged and is constitutively expressed and present in the cytoplasm of neurons. To activate ATF6 signaling, sATF6-KI mice received a 5-day tamoxifen treatment, which triggered nuclear translocation of sATF6, leading to ATF6-dependent gene expression (Supplementary Figure S1, Yu et al., 2017; Shen et al., 2021). Of note, Emx1^Cre/+^ littermate mice were used as controls. Control mice were treated with tamoxifen following the same procedure.

### Stroke models

#### Photothrombotic stroke

Photothrombotic stroke was performed as previously described (Wang et al., 2021). Briefly, after anesthesia induction (5% isoflurane), mice were placed in a stereotactic frame, and isoflurane anesthesia (1.5%–2%) was maintained using a face mask. Rectal temperature was maintained at 37°C ± 0.5°C using a feedback-controlled heating pad. After sterilizing the head skin with iodine and a 70% alcohol swab, a midline incision was made to expose the bregma. The Rose Bengal solution (Sigma-Aldrich, Saint Louis, MO; 10 mg/ml in saline) was intraperitoneally injected in a dose of 10 μl/g of body weight. After 5 min, a 3-mm diameter illumination (green light, SCHOTT KL1600) was positioned at the right cortex, and the center was 1.75 mm lateral to bregma. The brain was illuminated through the intact skull for 15 min.

#### Transcranial permanent MCAO

Permanent MCAO (pMCAO) was performed essentially the same as described previously (Jiang et al., 2017; Li et al., 2022). Briefly, mice were anesthetized with isoflurane, intubated, and ventilated. Rectal temperature was maintained at 37°C ± 0.2°C throughout the procedure. After a small neck midline incision was made, the right common carotid artery (CCA) was isolated and ligated using a 7–0 silk suture. Mice were then placed in a left lateral position, and a small transverse incision was made between the eye and ear to reveal the zygomatic arch. After removing a 3-mm segment of the zygomatic arch, and exposing the skull base and trigeminal nerve branch, a small window (1–2 mm^2^) was drilled in the skull above the MCA. The MCA trunk was then permanently ligated with a silk suture proximal to the cortical branch to the rhinal cortex.

### Drug administrations

Tamoxifen (Cayman Chemical, Ann Arbor, MI; 20 mg/ml) was suspended in corn oil (Sigma-Aldrich). Mice were treated with 100 mg/kg tamoxifen by oral gavage once daily for 5 days. The stroke surgery was performed 2 days after tamoxifen treatment. In the long-term study, we resumed tamoxifen treatment at the same dose beginning on day 4 poststroke and continuing once every other day thereafter until 15 days after stroke.

### Behavioral tests

All tests were performed in a blinded manner. Reference data for behavioral tests were presented in Supplementary Figure S2.

#### Neurologic score

This 48-point scoring system was used to evaluate neurologic deficits after stroke, and has been described in detail previously (Taninishi et al., 2016). Briefly, this relatively comprehensive system uses various assessments related to the general status, simple motor deficits, complex motor deficits, and sensory deficits. The final score for each animal was the sum of the scores for all assessments, with 0 = no deficit and 48 = maximal deficit.

#### Foot fault test

This test was performed as previously described, with slight modifications (Balkaya et al., 2013). Post-stroke animals were placed on an elevated grid (28L× 19W× 14H cm) with squares sized about 1.4 cm^2^. Forelimbs falling through the wire grid were recognized as foot faults. The number of foot faults made by ipsilateral and contralateral forelimbs and normal steps were recorded for 3 min. The foot fault test score was obtained as a percentage: (impaired forelimb slip − non-impaired forelimb slip)/(impaired forelimb slip + non-impaired forelimb slip + normal steps) × 100%.

#### Cylinder test

The cylinder test was used to evaluate limb use asymmetries in mice (Balkaya et al., 2013). Mice were placed in a glass cylinder (diameter: 9–10 cm; height: 15 cm) with a mirror behind the cylinder, and were video-recorded. Forelimb contacts while rearing were scored with a total of 20 contacts recorded for each animal. The asymmetry score was obtained as a percentage: (non-impaired forelimb contact − impaired forelimb contact)/(non-impaired forelimb contact + impaired forelimb contact + both contacts) × 100%.

#### Open field test

Open field test measures the spontaneous locomotor activity of post-stroke mice in a novel environment. All mice were acclimated to the testing room for 30 min before the test. Each mouse was placed in the center of the open field chamber (50 × 50 × 50 cm), and its motion was recorded for a total of 10 min. The travel distance was calculated by an automated animal behavior analysis system (CleverSysTopscan 3.0).

#### Tight rope test

This test was performed as previously described (Wang et al., 2020). Each mouse was placed with its forepaws on the middle of the rope, and the time lapse for the mouse to reach the platform was recorded. The maximal testing time was 60 s. Each test had two trials, and the average time was calculated.

#### Tape removal test

The adhesive tape removal test was used to evaluate sensory-motor impairments, following a published protocol with slight modifications (Bouet et al., 2009). Mice were pre-trained with one trial per day for 4–5 days before surgery. During the test, a small adhesive patch (0.3 × 0.4 cm) was attached to each forepaw. Each mouse was then placed in a transparent box, and observed for 2 min. The time-to-contact and the time-to-remove each patch were recorded.

### Laser speckle contrast imaging (LSCI)

LSCI was performed using a full-field laser perfusion imager RFLS (RWD Life Science Co, Kent, DE, USA), as described previously (Wang et al., 2021). A midline scalp incision was made to expose the skull, and the imager was positioned directly above the skull.

### Lectin labeling

Lectin labeling was performed as previously reported (Robertson et al., 2015). Under isoflurane anesthesia, mice were injected with 100 μl saline solution containing 50 μg DyLight- 594 lectin (Vector Laboratories, Newark, CA) *via* the right jugular vein. After 15 min, mice were transcardially perfused with saline and 4% paraformaldehyde. Mouse brains were collected, post-fixed with 4% paraformaldehyde for 24 h, immersed in 30% sucrose in PBS for 2 days, and then stored at −80°C. Frozen brain sections (35 μm thick) were obtained using a Leica cryostat. Images were captured on a Zeiss Axio Imager Z2 motorized fluorescence microscope (Carl Zeiss MicroImaging).

### Infarct volume

Infarct volume was assessed using the 2,3,5-triphenyltetrazolium chloride (TTC) staining method (Wang et al., 2021). In brief, brains were coronally sliced using a mouse brain matrix (ASI Instruments, Warren, MI). Brain sections (1 mm thick) were stained with 2% TTC, and then fixed in 10% formaldehyde. The TTC-stained brain sections were imaged and analyzed in a blinded fashion using ImageJ software (NIH). The infarct volume was calculated by subtracting the non-infarcted area in the ipsilateral hemisphere from the total area of the contralateral hemisphere.

### Western blotting

Western blot analysis was performed as described previously (Liu et al., 2016). Briefly, brain samples were homogenized by sonication using 2% SDS lysis buffer. Protein samples were run on SDS-PAGE gels and were transferred to PVDF membranes. After blocking, membranes were incubated with a primary antibody overnight at 4°C. After washing and incubation with a secondary antibody, proteins were then detected with the enhanced chemiluminescence analysis system (Cytiva, Marlborough, MA; GE Healthcare). β-actin was used as the loading control. The primary antibodies included FLAG (F3165; Sigma), GRP78 (610978; BD Biosciences), and β-actin (A3854; Sigma).

### Immunofluorescence analysis

Immunofluorescence staining was performed on frozen tissues, as described previously (Jiang et al., 2017). In short, mouse brains were fixed by transcardial perfusion with 4% paraformaldehyde, and stored at −80°C. Frozen brain sections (35 μm thick) were obtained using a Leica cryostat, and immunostained using a free-floating staining method with the following primary antibodies: rabbit anti-GFAP (glial fibrillary acidic protein; 1:500; Millipore, Burlington, MA, USA) and rabbit anti-Iba1 (ionized calcium-binding adaptor molecule 1; 1:500; Wako, Richmond, VA, USA). Images were captured on a Zeiss Axio Imager Z2 motorized fluorescence microscope (Carl Zeiss MicroImaging). The striatal areas of the ipsilateral and contralateral hemispheres were measured and calculated as the percentage of ipsilateral/contralateral striatal area. GFAP^+^ astrocytes, Iba1^+^ microglia, and lectin-stained vessels were analyzed in a blinded fashion by measuring fluorescence intensity in four defined regions of interest (ROI) in the ipsilateral or contralateral cortex and striatum (size: 500 × 500 μm; ROI centered 1.75 mm lateral to midline/0.75 mm, 2.5 mm, 3.5 mm, and 4.0 mm below brain surface). The mean values of GFAP and Iba1 fluorescence intensity and vascular density were calculated as the percentage of ipsilateral/contralateral ROI.

#### Statistical analysis

All data analyses were performed using Prism 8 (GraphPad Software, San Diego, CA). Infarct volume (short-term) or neurologic score (long-term) was used to determine the group size for each experiment. Statistical analysis was assessed by unpaired Student’s *t*-test (infarct volumes, tight rope, and fluorescence intensities), Mann-Whitney U test (neurologic scores), or by two-way ANOVA with Holm-Sidak post-hoc (open field, cylinder test, foot fault, and tape removal test). Data are presented as mean ± SD or the median (neurologic score), as shown in the figures. The level of significance was set at *p* < 0.05.

## Results

### Stroke outcome after PT stroke in sATF6-KI mice

The PT stroke model has been widely used in experimental stroke research due to the simplicity of the procedure and excellent long-term survival rates. Since deficits in motor function are common in stroke patients, our PT model targets the right M1 primary motor cortex region, which leads to left limb movement disorder in the mouse.

**Figure 1:**
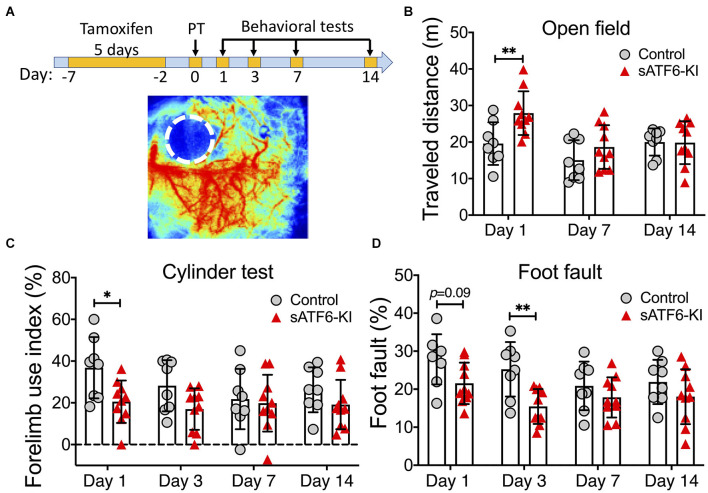
Activation of the ATF6 pathway in neurons improved short-term but not long-term outcome after photothrombotic stroke. **(A)** Experimental design. A representative laser speckle contrast image of PT stroke brain is shown below. **(B–D)** Functional outcome. sATF6-KI (*n* = 10) and littermate control (*n* = 8) mice were subjected to photothrombotic (PT) stroke. Behavioral tests, including open field **(B)**, cylinder **(C)**, and foot fault **(D)** tests, were performed over 14 days after PT stroke. Data are presented as mean ± SD. *p < 0.05; **p < 0.01.

For five consecutive days, sATF6-KI and littermate control mice were orally treated with tamoxifen to induce translocation of sATF6-MER from the cytosol into the nucleus where ATF6-dependent genes are then activated (Yu et al., 2017). Two days later, mice were subjected to PT stroke, followed by three behavioral tests for up to 2 weeks post stroke (Figure 1A). On day 1 after stroke, sATF6-KI mice exhibited a significantly longer travel distance in the open field test, more balanced use of both forelimbs in the cylinder test, and fewer errors in the foot fault test compared to control mice (Figures 1B–D). On day 3 after PT stroke, sATF6-KI mice continued to exhibit better performance. However, on post-stroke day 7 and thereafter, no significant difference in performance was observed between groups. This observation was not completely unexpected, because in our PT stroke model, only a small cortical infarct was produced, and motor functions may largely recover in stroke mice. Since stroke outcome beyond the acute phase is critical, we decided to apply a more severe permanent stroke model.

### Acute stroke outcome after permanent MCAO (pMCAO) in sATF6-KI mice

**Figure 2:**
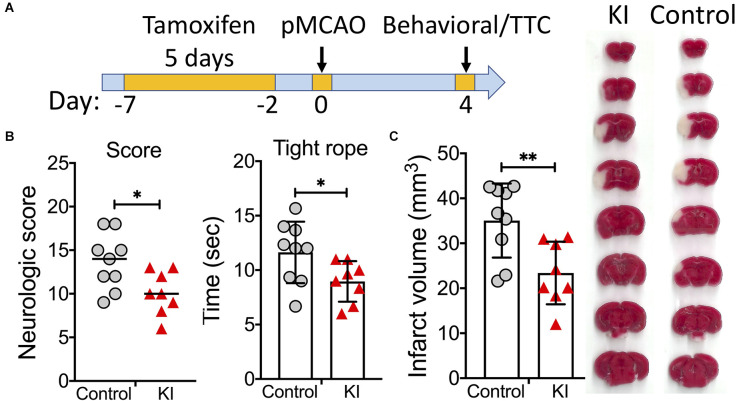
Activation of the ATF6 pathway in neurons improved short-term outcome after permanent MCAO (pMCAO). **(A)** Experimental design. **(B,C)** Short-term stroke outcome. sATF6-KI (KI; *n* = 8) and littermate control (*n* = 9) mice were subjected to pMCAO. Neurologic functions, including neurologic scoring and tight rope test, were evaluated on day 4 after stroke **(B)**. Then, infarct volumes were measured **(C)**. Representative TTC-stained brain sections are shown. Data are presented as mean ± SD or median. *p < 0.05; **p < 0.01.

Our transcranial permanent MCAO model was chosen because this model produces a relatively large infarct that extends into both the cortex and striatum, and still has an excellent long-term survival rate. We first performed a short-term outcome study using this model. On day 4 after pMCAO, functional deficits and infarct volumes were evaluated (Figure 2). Compared to control mice, sATF6-KI mice performed significantly better in both neurologic scoring and tight rope test (Figure 2B). Consistent with behavioral data, sATF6-KI mice also exhibited smaller infarcts (Figure 2C). Together with our previous study (Yu et al., 2017), we have provided evidence that activation of the ATF6 pathway in neurons offers acute protective effects in both transient and permanent MCAO models.

### Long-term stroke outcome after pMCAO in sATF6-KI mice with chronic activation of the ATF6 pathway

**Figure 3:**
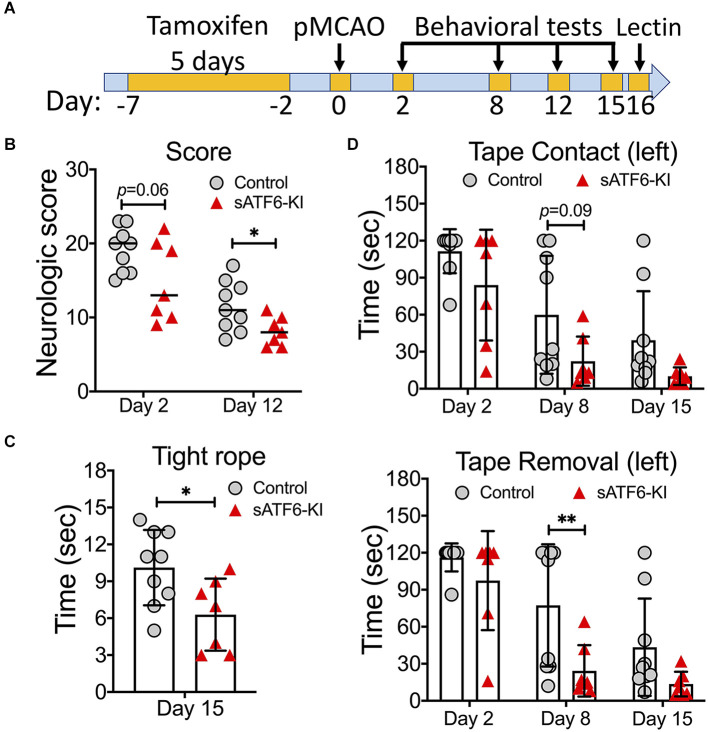
Activation of the ATF6 pathway in neurons improved long-term outcome after permanent MCAO (pMCAO). **(A)** Experimental design. **(B–D)** Stroke outcome. sATF6-KI (*n* = 7) and littermate control (*n* = 9) mice were subjected to pMCAO. Of note, all mice received tamoxifen treatment every other day from 4 to 15 days after stroke behavioral tests, including neurologic scoring **(B)**, tight rope test **(C)**, and tape removal test **(D)**, were performed over 15 days after stroke. Data are presented as mean ± SD or median. *p < 0.05; **p < 0.01.

Next, we set out to evaluate the stroke outcome in sATF6-KI mice beyond the acute phase, i.e., during a period of 15 days after stroke (Figure 3). Since tamoxifen-induced sATF6 nuclear translocation is transient, we extended the effects of ATF6 activation by dosing tamoxifen every other day from day 4 to day 15 after stroke. Compared to control mice, sATF6-KI mice consistently performed better on neurologic scoring (Figure 3B), tight rope test (Figure 3C), and tape removal test (Figure 3D). Although mice from both groups exhibited functional recovery over time, sATF6-KI mice appeared to recover to a greater extent.

### Brain pathology after pMCAO in sATF6-KI mice

**Figure 4:**
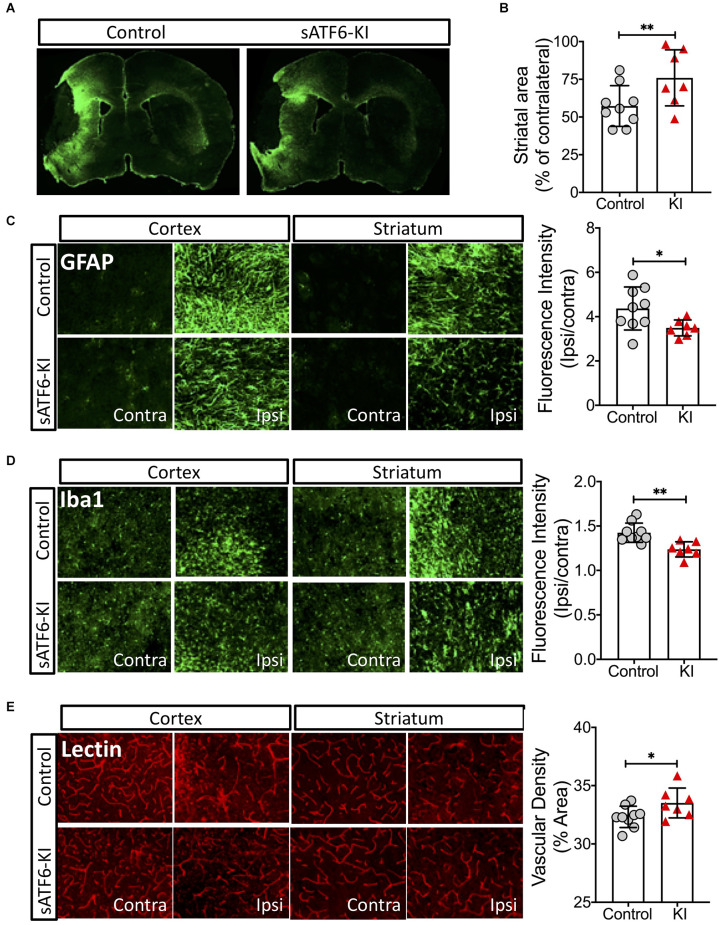
Effects of neuronal ATF6 activation on the brain after pMCAO. The mice were from the same experiment as in Figure 3. On day 16 after stroke, all mice received lectin injection before euthanasia. Brain sections were used for analyses. **(A–C)** Brain sections were stained with GFAP (an astrocyte marker). Representative GFAP-stained coronal brain sections **(A)**. Striatal volumes **(B)**. Astrogliosis **(C)**. Microglia activation was evaluated by Iba-1 staining **(D)**. Vascular density was evaluated by lectin signal **(E)**. Data are presented as mean ± SD. *p < 0.05; **p < 0.01. Contra, Contralateral; Ipsi, Ipsilateral; KI, sATF6-KI.

On day 16 post stroke, all mice were subjected to lectin labeling and then, euthanized. Brain sections were stained with GFAP (an astrocyte marker) or Iba1 (a microglia marker). Using GFAP-stained brain sections, we found that sATF6-KI mice had significantly larger striatal areas than control mice, indicating less striatal atrophy (Figures 4A,B). As expected, a large number of reactive astrocytes was present in the peri-infarct region. Measurement of GFAP fluorescence intensity indicated less astrogliosis in sATF6-KI mouse brains (Figure 4C). Our data also showed less neuroinflammation in sATF6-KI mice, based on Iba1 staining data (Figure 4D). Finally, using lectin signal, we found that compared to control mice, sATF6-KI mice had significantly higher levels of peri-infarct vascular density (Figure 4E), suggesting better preservation of cerebral vasculature in sATF6-KI mice.

## Discussion

Here, we performed the first experimental study to demonstrate that activation of the ATF6 pathway improves not only the acute outcome but also long-term outcome after permanent ischemic stroke.

Proteins are the key biologic molecules that perform most of the work in cells. Thus, proteostasis is essential to cell health and survival. Indeed, misfolded or damaged proteins are dysfunctional, and are prone to form aggregates, which can be toxic and cause cell death. To maintain or restore proteostasis, a complex protein quality control network functions in subcellular components that are involved in the biogenesis of proteins, especially the cytoplasm and ER. In the cytoplasm, heat shock proteins (HSPs) are the major protein chaperones. Under physiologic conditions, HSPs fold newly synthesized proteins, while under stress conditions, they are required to prevent protein aggregation, mediate protein disaggregation, and remove dysfunctional proteins and protein aggregates. Among these HSPs, cytosolic HSP70 plays a vital role in restoring proteostasis in pathologic states (Rosenzweig et al., 2019). In the ER, the most abundant protein chaperon is GRP78, which is also a member of the HSP70 family. A large body of evidence has indicated that GRP78 in the ER is functionally equivalent to cytosolic HSP70. Like HSP70, GRP78 is involved in protein disaggregation, and increased expression of GRP78 markedly reduces protein aggregation in the ER (Melo et al., 2022). Notably, evidence has suggested that an increase in GRP78 expression is protective in stroke (Ouyang et al., 2012). In sATF6-KI mice, GRP78 expression is markedly increased (Supplementary Figure S1), as GRP78 expression is primarily regulated by ATF6 transcriptionally (Yu et al., 2017). Thus, GRP78-mediated restoration of cellular proteostasis may play a major role in improving stroke outcome in sATF6-KI mice (Yu et al., 2017).

Since sATF6 is a potent transcription factor, many proteins, in addition to GRP78, may contribute to this protective effect. For example, our RNA-seq analysis has indicated that a number of key ERAD components can be upregulated by sATF6, including VCP, HERP, and HRD1 (Shen et al., 2021). ERAD is an evolutionarily conserved pathway that is specifically responsible for clearing misfolded proteins in the ER by eventually using the ubiquitin-proteasome system (UPS) in the cytosol (Wiseman et al., 2022). The main steps of ERAD include ubiquitination and retro-translocation of misfolded proteins from the ER into the cytosol where they can enter the UPS for degradation. This process is primarily mediated by an ERAD complex in which HRD1, an E3 ligase, is a key component for ubiquitination, VCP assists in extracting ubiquitinated ER proteins into the cytosol, and HERP functions as a regulatory component. In general, current evidence supports the notion that upregulation of ERAD-related genes is a pro-survival process under stress conditions. For example, deletion of HERP significantly increases infarct volumes in mice after permanent stroke (Eura et al., 2012). Overexpression of HRD1 in myocytes preserves cardiac function in mice subjected to pressure overload (Doroudgar et al., 2015). Thus, it is likely that augmented ERAD capacity in neurons contributes to the improved stroke outcome observed in sATF6-KI mice.

Using the conditional and tamoxifen-inducible sATF6 knock-in mouse line sATF6-MER that was developed by our group, we previously showed that ATF6 activation in neurons provided acute beneficial effects after transient ischemic stroke (Yu et al., 2017), supporting the notion that the ATF6 pathway is a potential therapeutic target in stroke. Our data in the current study offer critical further support for this notion by investigating both short- and long-term stroke outcomes after permanent stroke. Of note, an improvement in short-term, but not long-term, outcome was observed in sATF6-KI mice if a PT stroke model was used. This is likely due to the fact that after PT stroke, there is only a small infarct in the cortex with relatively little salvageable penumbral tissue (Carmichael, 2005). It is known that neurologic functions impaired initially by stroke can gradually and partially restore over time, which makes it challenging to detect the long-term effects of a treatment using a PT stroke model. In our case, more sensitive behavioral tests may be required.

Overall, our data align well with studies from other groups. For example, ATF6α knockout mice exhibited larger infarct volumes after transient MCAO (Yoshikawa et al., 2015). Pharmacologic increase of GRP78 can reduce both infarct volumes and brain swelling in mice after permanent MCAO (Oida et al., 2010). Notably, using an ATF6-specific inducer (i.e., compound 147), Blackwood et al. found that activation of the ATF6 pathway exerts protective effects in various mouse ischemia/reperfusion models including heart ischemia, renal ischemia, and ischemic stroke, demonstrating that ATF6 signaling is likely a universal pro-survival pathway that protects cells from ischemic injury (Blackwood et al., 2019).

Collectively, we now have evidence that activation of ATF6 signaling improves outcomes in both transient and permanent ischemic stroke. Thus, rigorous preclinical studies are warranted to further evaluate the therapeutic potential of targeting the ATF6 pathway in ischemic stroke. To this end, ATF6-specific activators that can cross the blood–brain barrier are key. In this respect, high throughput drug screening has led to initial success by identifying compound 147. However, the effect of compound 147 on GRP78 upregulation appears to be modest in the brain compared to peripheral organs (Blackwood et al., 2019), and its pharmacokinetic properties in the brain remain largely unknown. Therefore, to advance translational research on targeting the ATF6 pathway in stroke, new potent and well-characterized ATF6-specific activators are still required.

## Data Availability Statement

The original contributions presented in the study are included in the article/Supplementary material, further inquiries can be directed to the corresponding author.

## Ethics Statement

The animal study was reviewed and approved by Duke IACUC.

## Author Contributions

XL and WY designed research and wrote the manuscript. XL, HS, and WY analyzed data. XL, RL, LL, AD, and HS performed research. All authors contributed to the article and approved the submitted version.

## Funding

This study was supported by funds from the Department of Anesthesiology (Duke University Medical Center) and NIH grants NS099590 and HL157354.
