# Supplementary material for: Beneficial effects of neuronal ATF6 activation in permanent ischemic stroke
Source: Front Cell Neurosci. 2022 Oct 14;16:1016391. doi: 10.3389/fncel.2022.1016391 (PMC9614111; doi:10.3389/fncel.2022.1016391)
Supplement: Supplementary file 1 [file Data_Sheet_1.PDF]

Supplemental Material

Beneficial effects of neuronal ATF6 activation in permanent ischemic stroke

Xuan Li, Ran Li, Liping Lu, Ashis Dhar, Huaxin Sheng, Wei Yang

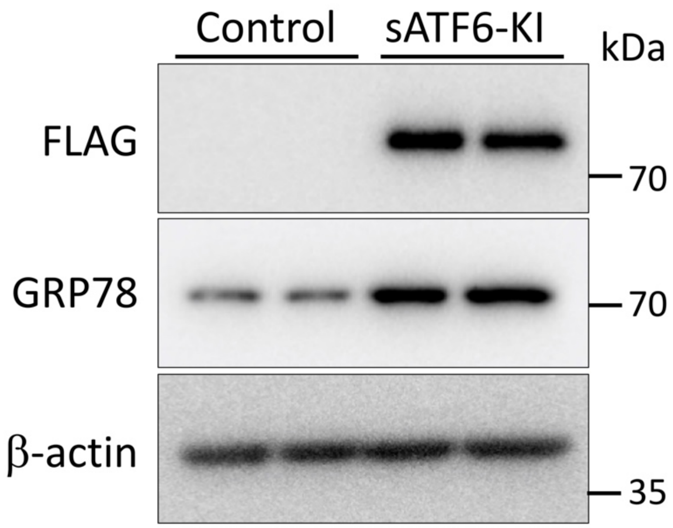

Figure S1. Confirmation of FLAG-sATF6-MER transgene expression and upregulation of GRP78 in sATF6-KI mouse brains. Littermate control and sATF6-KI mice were treated with tamoxifen for 5 days. Two days later, the brain cortex tissue samples were examined by Western blotting.

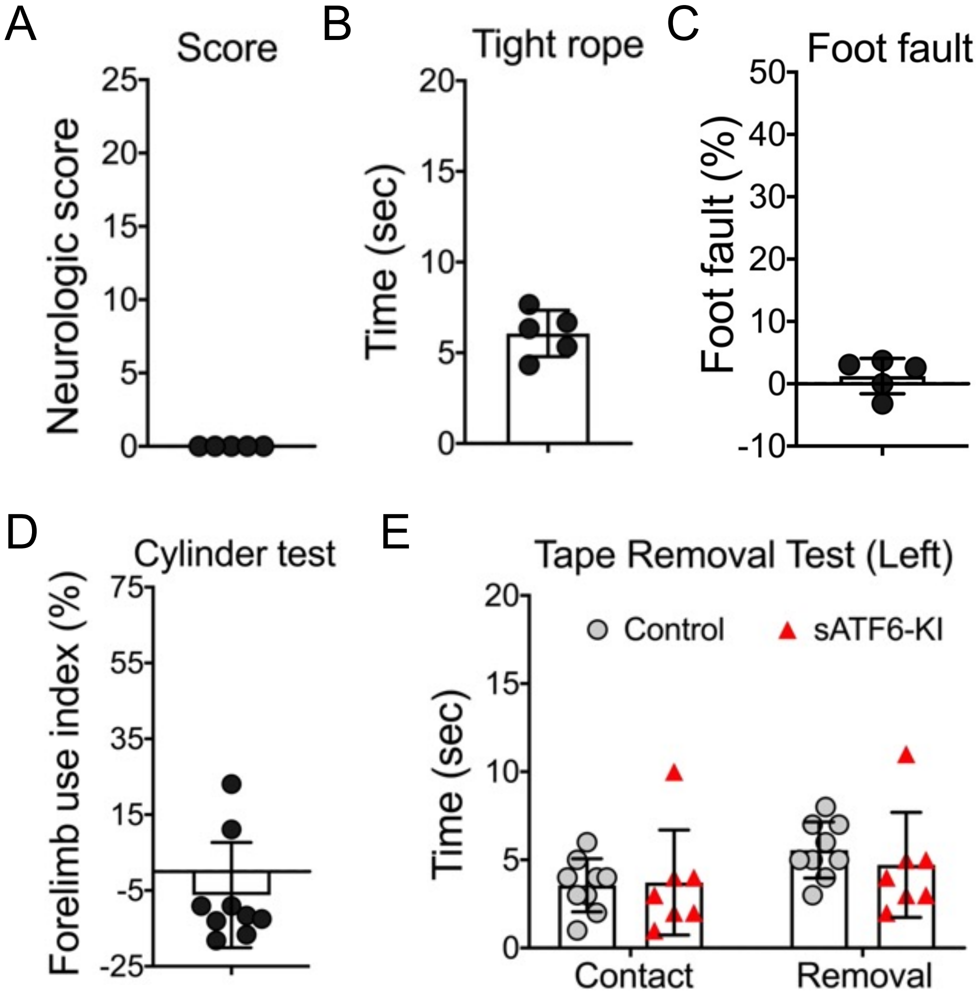

Figure S2. Baselines of behavioral tests. Mice were subjected to various behavioral tests to establish the baselines before surgery. The data of neurologic scoring (A), tight rope (B), foot fault test (C), and cylinder test (D) were generated in a pilot study (n = 5-9; C57Bl/6 mice), while the data in tape removal test (E) were collected before stroke surgery (n = 7-9; control vs sATF6-KI in Fig. 3). For the purpose of calculation in D, the left side was considered as impaired.
